# Full-length SMRT transcriptome sequencing and microsatellite characterization in *Paulownia catalpifolia*

**DOI:** 10.1038/s41598-021-87538-8

**Published:** 2021-04-22

**Authors:** Yanzhi Feng, Yang Zhao, Jiajia Zhang, Baoping Wang, Chaowei Yang, Haijiang Zhou, Jie Qiao

**Affiliations:** 1 Paulownia Research and Development Center of State Administration of Forestry and Grassland, Zhengzhou, 450003 China; 2 Non-Timber Forestry Research and Development Center, Chinese Academy of Forestry, Zhengzhou, 450003 China; 3 Key Laboratory of Non-Timber Forest Germplasm Enhancement and Utilization of State Forestry Administration, Zhengzhou, 450003 China; 4 National Innovation Alliance of Paulownia, Zhengzhou, 450003 China

**Keywords:** Molecular biology, Plant sciences

## Abstract

*Paulownia catalpifolia* is an important, fast-growing timber species known for its high density, color and texture. However, few transcriptomic and genetic studies have been conducted in *P. catalpifolia*. In this study, single-molecule real-time sequencing technology was applied to obtain the full-length transcriptome of *P. catalpifolia* leaves treated with varying degrees of drought stress. The sequencing data were then used to search for microsatellites, or simple sequence repeats (SSRs). A total of 28.83 Gb data were generated, 25,969 high-quality (HQ) transcripts with an average length of 1624 bp were acquired after removing the redundant reads, and 25,602 HQ transcripts (98.59%) were annotated using public databases. Among the HQ transcripts, 16,722 intact coding sequences, 149 long non-coding RNAs and 179 alternative splicing events were predicted, respectively. A total of 7367 SSR loci were distributed throughout 6293 HQ transcripts, of which 763 complex SSRs and 6604 complete SSRs. The SSR appearance frequency was 28.37%, and the average distribution distance was 5.59 kb. Among the 6604 complete SSR loci, 1–3 nucleotide repeats were dominant, occupying 97.85% of the total SSR loci, of which mono-, di- and tri-nucleotide repeats were 44.68%, 33.86% and 19.31%, respectively. We detected 112 repeat motifs, of which A/T (42.64%), AG/CT (12.22%), GA/TC (9.63%), GAA/TTC (1.57%) and CCA/TGG (1.54%) were most common in mono-, di- and tri-nucleotide repeats, respectively. The length of the repeat SSR motifs was 10–88 bp, and 4997 (75.67%) were ≤ 20 bp. This study provides a novel full-length transcriptome reference for *P. catalpifolia* and will facilitate the identification of germplasm resources and breeding of new drought-resistant *P. catalpifolia* varieties.

## Introduction

*Paulownia*, one of the most important fast-growing timber species around the world, is native to China and widely grown in subtropical and warm temperate regions, which have acted an important part of timber supply, ecological environmental construction, soil improvement and so forth^1^. *Paulownia catalpifolia* is a typical and important species of Genus Paulownia in northern China, it exhibits some drought resistance and is renowned for its high density, good color, and beautiful texture. Recently, droughts and water shortages have seriously affected *P. catalpifolia* growth, causing mass deaths in some *P. catalpifolia* plantations. Therefore, high-quality and drought-resistant *P. catalpifolia* varieties are urgently needed. Conventional plant breeding methods, such as cross-breeding and selection breeding, have yielded little success in improving the traits of plants; this is due to genetic resistance, reproductive isolation and long generation cycles, among other factors^2,3^. Previous studies on *P. catalpifolia* have focused on the chemical composition of its fruits and seeds, as well as on tissue culture^4–6^; however, molecular studies of *P. catalpifolia* are lacking.

Microsatellites, also known as simple sequence repeats (SSRs), are DNA sequences consisting of continuously repeating motifs, which are composed of 1–6 bases^7,8^. The type and number of repeat motifs differ among SSRs, resulting in polymorphisms at each SSR locus. SSR molecular markers are widely distributed throughout plant genomes^9^ and are characterized by codominance, high polymorphism and good repeatability. SSR loci are conserved within and among genera^10^. Depending on their origin, SSR markers can be categorized as genomic SSRs or expressed sequence tag (EST) SSRs. EST-SSR markers are easier to obtain for a large number of plants that have no reference genome, although the polymorphism of EST-SSR is lower than that of genomic SSR markers. As functional molecular markers, EST-SSRs are more conserved, better universality, lower cost and more interspecific transferability^11,12^. Moreover, EST-SSR polymorphisms may be directly related to gene function^13^ and can be used for researches of other related species^14,15^. In recent years, EST-SSR markers have been developed and applied in various tree species, including *Eucalyptus globulus*^16^, *Euphrates Poplar*^17^, the rubber tree^18^, *Robinia pseudoacacia*^19^, *Fraxinus velutina*^20^, and *Pinus koraiensis*^21^.

Single-molecule real-time (SMRT) sequencing technology (Pacific Biosciences), also known as third generation sequencing technology, can efficiently and accurately obtain high-quality (HQ), long and intact transcripts containing 5′- and 3′-untranslated regions and polyadenosine tails without assembly^22,23^. SMRT sequencing can be used to accurately identify features such as fusion genes, gene families, long non-coding RNAs (lncRNAs) and alternative splicing (AS) events^24,25^. SMRT sequencing technology is a reliable method for obtaining full-length transcripts that can be used to study the transcriptomes of non-model plants which lack reference genomes, such as *Paulownia* and Chinese *catalpa*. SMRT sequencing technology has been successfully applied to full-length transcriptome sequencing studies in animals, plants and insects^26–28^. Furthermore, full-length transcriptome sequences obtained using SMRT sequencing contain numerous EST SSRs^29,30^, which can be used for genetic analyses of the sequenced species and their related species, as well as for studies of conservation biology and molecular assisted breeding^23,31,32^. To the best of our knowledge, no full-length transcriptome sequence of *P. catalpifolia* has been reported.

In this study, we performed a full-length transcriptomic analysis of mixed *P. catalpifolia* leaves treated with varying degrees of drought stress using SMRT sequencing. We then performed function annotation analyses using publicly available databases and used various bioinformatics software to predict AS, lncRNAs and SSRs and to further analyze SSRs characteristics deeply. In the absence of Paulownia reference genome, the full-length transcriptome sequence acquired in our study not only can be used as a reference sequence for transcriptome sequencing, but also will support further genetic analyses in *Paulownia* species. In addition, the SSRs predicted in our study will facilitate the development of drought-resistant SSR markers, the discovery of drought-resistant genes and the study of the genetic relationships between *P. catalpifolia* and other related species.

## Results

### SMRT sequencing of the full-length transcriptome

**Figure 1:**
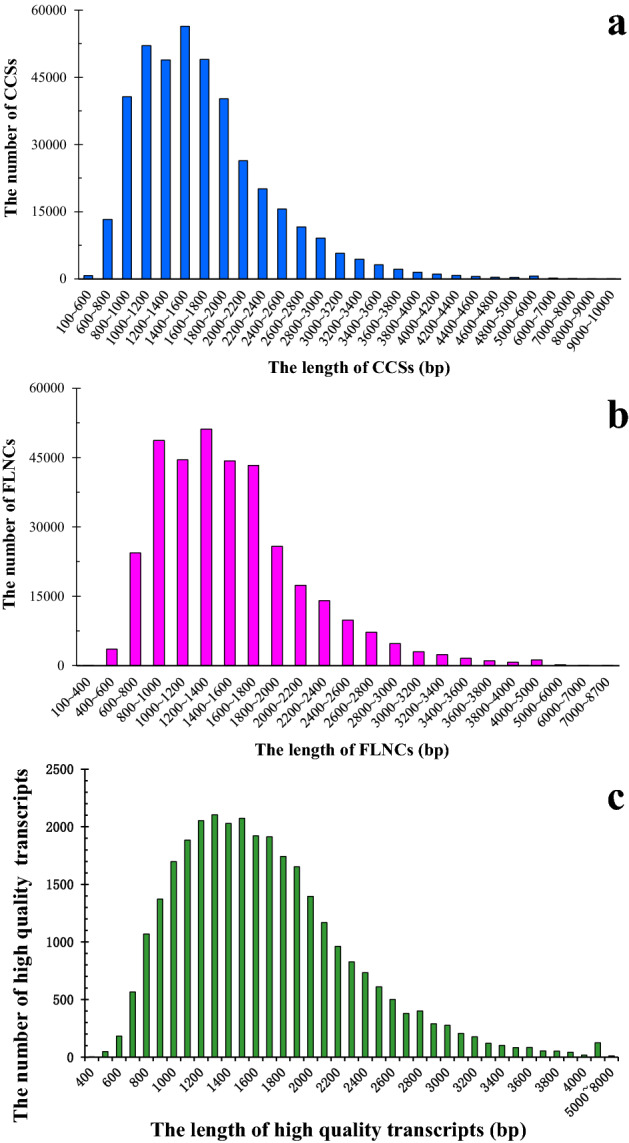
SMRT sequencing of *P. catalpifolia* leaf transcriptomes. (**a**) Length distribution of CCSs. (**b**) Length distribution of FLNCs. (**c**) Length distribution of high-quality transcripts. Figure was made by Microsoft Office Excel 2013 software.

We acquired full-length transcriptomic of *P. catalpifolia* using SMRT sequencing technology and obtained 28.83 Gb sequencing data. After removing the adapter sequences, approximately 454,554 polymerase reads remained, which then formed 19,052,345 subreads with an average read length of 1470 bp. After self-correction and merging, the subreads formed 405,034 circular consensus sequences (CCSs) (Fig. 1a) with an average length of 1693 bp, and 349,745 full-length non-chimeric sequences (FLNCs) (Fig. 1b). A total of 30,953 transcripts were obtained after clustering and removal of redundant sequences using the PacBio SMRT LINK Cluster tool, and 30,928 HQ transcripts with ≥ 99% accuracy and a full-length read support ≥ 2 were sequenced (Fig. 1c). The length range of the HQ transcripts was 362–7922 bp, the N50 was 1768 bp, and the mean transcript length was 1618 bp. Of the HQ transcripts, 10.47% and 86.07% were 362–900 bp and 1000–3000 bp in length, respectively. Long-length HQ transcripts (> 3000 bp) constituted 3.46% of the total HQ transcripts. After error correction and removal of all 100% identical sequences, 25,969 HQ transcripts remained, its individual transcript length ranging from 362 to 7922 bp, the average length of 1624 bp, and N50 of 1781 bp, which were used in subsequent analyses.

### Functional annotation of the full-length transcriptome sequences

**Figure 2:**
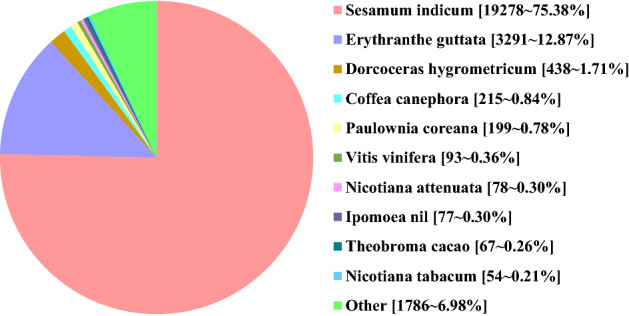
The Homologous species distribution of *P. catalpifolia* HQ transcripts. Figure was made by Microsoft Office Excel 2013 software.

**Figure 3:**
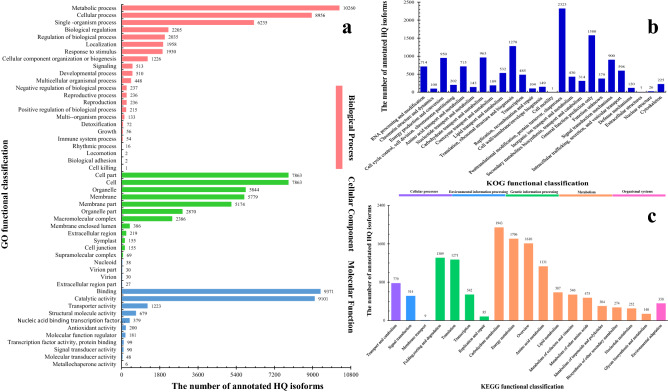
Gene ontology (GO), eukaryotic orthologous groups (KOG) and Kyoto encyclopedia of genes and genomes (KEGG) functional classifications of high-quality (HQ) transcripts. (**a**) GO classification of HQ transcripts. (**b**) KOG classification of HQ transcripts. (**c**) KEGG classification of HQ transcripts.

The functional annotation of the HQ transcripts was then performed. Of the 25,969 transcripts analyzed, 367 could not be functionally assigned by any of the databases used (Table 1). A total of 25,591 (98.54%) HQ transcripts were annotated using the NCBI non-redundant protein database and exhibited homology with known proteins of various species, including *Sesamum indicum* (75.38%), *Erythranthe guttata* (12.87%) and *Dorcoceras hygrometricum* (1.71%) (Fig. 2). The HQ transcripts were then searched against the gene ontology (GO) database to analyze their functions; 18,501 (71.24%) of the HQ transcripts were categorized into 50 GO group, which were divided into three broad classes: biological processes (37,536 HQ transcripts, 38.38%), cellular components (38,888, 39.76%) and molecular functions (21,377, 21.86%) (Fig. 3a). Following searches against the eukaryotic orthologous groups (KOG) database, the HQ transcripts were clustered into 26 KOG terms (Fig. 3b). Furthermore, 13,829 HQ transcripts were identified in the Kyoto encyclopedia of genes and genomes (KEGG) database and grouped into 129 KEGG pathways, which were divided into five broad categories: cellular processes (779 HQ transcripts, 5.63%), environmental information processing (523, 3.78%), genetic information processing (3207, 23.19%), metabolism (8962, 64.81%) and organismal systems (358, 2.59%) (Fig. 3c). Using Swiss-Prot, 22,606 (87.05%) HQ transcripts were annotated.

**Table 1:** Results of the functional annotation of 25,969 HQ transcripts.

Database	Number of HQ transcripts	Percentage (%)
Annotated in NR	25,591	98.54
Annotated in GO	18,501	71.24
Annotated in KOG	12,350	47.56
Annotated in Swiss-Prot	22,606	87.05
Annotated in KEGG	13,829	53.25
Unannotated	367	1.41
Total HQ isoforms	25,969	100

### Identification of long non-coding RNAs, coding sequences and alternative splicing

**Figure 4:**
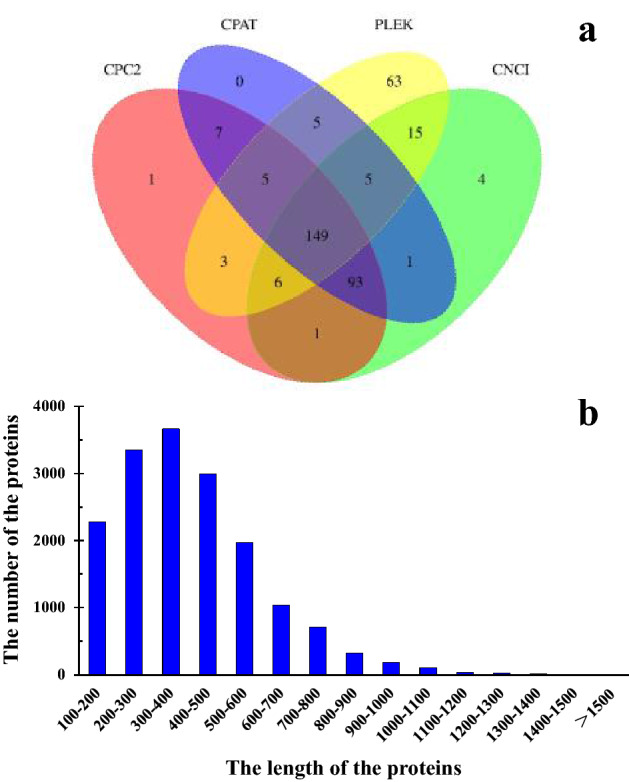
The identification of lncRNAs and the proteins length distribution of the *P. catalpifolia* transcriptome. (**a**) The Venn diagram of the number of lncRNAs predicted by CPC2, CPAT, PLEK and CNCI. (**b**) The length distribution of the proteins translated using predicted intact CDSs.

The long non-coding RNAs (lncRNAs) are not translated into protein and its length are more than 200 nucleotides. LncRNAs are vital for regulating the neighboring gene expression^33^. A total of 149 common lncRNAs were identified in *P. catalpifolia* Using four methods (CPC2, CPAT, PLEK and CNCI) (Fig. 4a). TransDecoder software was used to predict 24,982 coding sequences (CDSs), of which 16,722 were intact. The lengths of the amino acids encoded by the intact CDSs were in the range of 100–1840, with the number of amino acids decreasing as the length increased except 100–300 (Fig. 4b). Alternative splicing (AS) is one of crucial biological phenomenons, and it is helpful to produce different mature transcripts using the same RNA sequence^34^. AS is highly correlated with biological function and a major source of proteomic diversity. A total of 179 AS events were predicted without reference to genomic information in our research.

### Identification and characteristic analysis of SSRs

SSR loci were identified within the *P. catalpifolia* full-length transcriptome using MISA microsatellite software. A total of 7367 SSRs were identified, including 763 complex SSRs and 6604 complete SSRs. The total number of HQ transcripts containing SSRs was 6293, of which 747 contained ≥ 2 SSRs. SSRs occurred at a frequency of 24.23% (100% × total number of HQ transcripts containing SSRs/total number of HQ transcripts examined). The average distribution distance was 5.59 kb and the SSR appearance frequency was 28.37% (100% × total number of SSRs identified / total number of HQ transcripts examined) (Table 2).

**Table 2:** Occurrence of microsatellites in the full-length transcriptome of *P. catalpifolia.*

Item	Number
Total number of HQ transcripts examined	25,969
Total size of the examined HQ transcripts (bp)	42,183,906
Total number of HQ transcripts containing SSRs	6293
Total number of SSRs identified	7367
Total number of complex SSRs identified	763
Number of HQ transcripts containing more than one SSR	747

**Figure 5:**
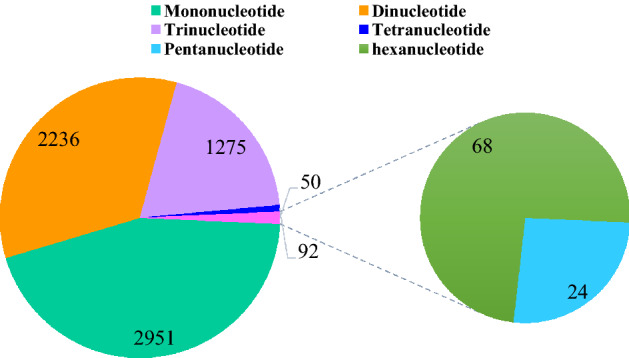
The types and numbers of complete SSRs in *P. catalpifolia.* Figure was made by Microsoft Office Excel 2013 software.

The number of complete SSRs was 6604 in total and accounted for 89.64% of the total SSR loci, which included 2951 mononucleotide (44.68%), 2236 dinucleotide (33.86%), 1275 trinucleotide (19.31%), 50 tetranucleotide (0.76%), 24 pentanucleotide (0.36%) and 68 hexanucleotide SSRs (1.03%) (Fig. 5). The complete SSR lengths ranged from 10 to 88 bp, with a mean of 15.99 bp. The number of repeat SSR motifs ranged from 5 to 44, with a mean of 10.03. We found that SSRs with 6 motif repeats were the most common and accounted for 13.64% (901) of all SSRs, followed by SSRs with 10 repeats (897, 13.58%), 5 repeats (834, 12.63%) and 11 repeats (757, 11.46%), respectively. Furthermore, 4997 SSRs had motif repeat numbers ≤ 12, accounting for 75.67% of all SSR loci identified (Table 3).

**Table 3:** The six types of SSR repeat motifs and their frequency in *P. catalpifolia.*

Repeat motif length	Repeat number									Total number	Frequency (%)
	5	6	7	8	9	10	11	12	> 12		
A/T						704	577	403	1132	2816	42.64
C/G						16	26	18	75	135	2.04
AC/GT		35	28	32	44	27	13	14	41	234	3.54
CA/TG		78	33	33	15	7	16	5	28	215	3.26
AG/CT		230	121	93	52	58	46	37	170	807	12.22
GA/TC		180	96	66	56	48	38	22	130	636	9.63
AT/AT		45	22	19	31	13	14	14	13	171	2.59
TA/TA		38	34	29	21	12	10	9	12	165	2.50
GC/GC			2	2						4	0.06
CG/CG		4								4	0.06
AAC/GTT	2	3		1	1					7	0.11
AAG/CTT	49	11	5	5	1	1	4	1	1	78	1.18
AAT/ATT	8	5	5	1		1	1			21	0.32
ACA/TGT	4	1								5	0.08
ACC/GGT	26	14	5	2	1					48	0.73
ACG/CGT	1	2	3							6	0.09
ACT/AGT	12	4		1						17	0.26
AGA/TCT	42	14	5	7	5	2	4	1	2	82	1.24
AGC/GCT	34	5	5	5	1					50	0.76
AGG/CCT	27	8	5	1						41	0.62
ATA/TAT	11	2	1	1					1	16	0.24
ATC/GAT	26	5	9	1		1				42	0.64
ATG/CAT	34	17	1	5	3					60	0.91
CAA/TTG	13	2	2	2	1					20	0.30
CAC/GTG	30	17	6	5		2				60	0.91
CAG/CTG	40	12	3	19	4		2		1	81	1.23
CCA/TGG	72	17	4	5			3	1		102	1.54
CCG/CGG	34	24	11	5	4					78	1.18
CGA/TCG	2	6								8	0.12
CGC/GCG	26	1	2	5		1				35	0.53
CTA/TAG	9	1								10	0.15
CTC/GAG	38	16	4		1		1			60	0.91
GAA/TTC	50	25	13	8	2	3	1	1	1	104	1.57
GAC/GTC	7	2	1							10	0.15
GCA/TGC	24	5	6	5						40	0.61
GCC/GGC	35	18		4		1				58	0.88
GGA/TCC	26	10	6	3	3					48	0.73
GTA/TAC	1	1								2	0.03
TAA/TTA	6	5	1	5			1			18	0.27
TCA/TGA	49	6	6	6				1		68	1.03
ATCA/TGAT			1							1	0.02
TTTG/CAAA	3									3	0.05
AAAT/ATTT	2	1								3	0.05
GGAA/TTCC				1						1	0.02
CCCT/AGGG		1	7							8	0.12
TTTA/TAAA	2									2	0.03
TGTA/TACA		1								1	0.02
TTCT/AGAA		2								2	0.03
ACAG/CTGT	2									2	0.03
GAAA/TTTC	2	1								3	0.05
TGAA/TTCA	4									4	0.06
TCTT/AAGA	1									1	0.02
ATGT/ACAT	1									1	0.02
CGTG/CACG	1									1	0.02
GATT/AATC	4									4	0.06
TCTA/TAGA					1					1	0.02
CTTT/AAAG	2	1								3	0.05
ATAC/GTAT				1						1	0.02
TTGT/ACAA		1								1	0.02
GCCC/GGGC	1									1	0.02
GGAG/CTCC	2	1								3	0.05
CAAC/GTTG	1									1	0.02
AATA/TATT	1									1	0.02
AAAC/GTTT	1									1	0.02
CCACC/GGTGG	9									9	0.14
TGATG/CATCA		1								1	0.02
TCCTC/GAGGA	2	2								4	0.06
CCACA/TGTGG	1									1	0.02
CTTTT/AAAAG	1	1								2	0.03
CACTT/AAGTG		1								1	0.02
TTCTT/AAGAA	1									1	0.02
TATTT/AAATA	1									1	0.02
CACCC/GGGTG	1									1	0.02
CCCAC/GTGGG	1									1	0.02
CTCTT/AAGAG	1									1	0.02
AGCTT/AAGCT	1									1	0.02
AAAAAG/CTTTTT	2									2	0.03
AAGAGA/TCTCTT	8									8	0.12
ACAGGG/CCCTGT		2								2	0.03
ACTCCG/CGGAGT	3									3	0.05
AGGAAA/TTTCCT		1								1	0.02
AGGAGA/TCTCCT	3									3	0.05
AGGCTC/GAGCCT		2								2	0.03
ATGGGC/GCCCAT		1								1	0.02
ATTTTC/GAAAAT		3								3	0.05
CACCAG/CTGGTG	2									2	0.03
CACCCC/GGGGTG	1									1	0.02
CACGCA/TGCGTG		1								1	0.02
CAGCAA/TTGCTG	1									1	0.02
CATCTT/AAGATG	1									1	0.02
CCATCT/AGATGG	2									2	0.03
CCCACT/AGTGGG	1									1	0.02
CCCTTT/AAAGGG	1									1	0.02
CCGCCA/TGGCGG	2	1								3	0.05
CCGGGA/TCCCGG	3									3	0.05
CCTCCC/GGGAGG	3									3	0.05
CCTCTC/GAGAGG	1									1	0.02
CCTCTT/AAGAGG	1									1	0.02
CTCAAC/GTTGAG	1									1	0.02
CTCCAC/GTGGAG		1								1	0.02
CTCCAT/ATGGAG	1		1							2	0.03
GAACCA/TGGTTC	2									2	0.03
GAGCCG/CGGCTC	2									2	0.03
GAGGAT/ATCCTC			1							1	0.02
GGAATG/CATTCC	1									1	0.02
GGAGCA/TGCTCC			1							1	0.02
GGTGGA/TCCACC	1									1	0.02
TCCGCC/GGCGGA	1									1	0.02
TCCTTT/AAAGGA	1									1	0.02
TTTCTT/AAGAAA		6								6	0.09
TTTTCT/AGAAAA	1									1	0.02
TTTTGC/GCAAAA	1									1	0.02
Total number	834	901	456	378	247	897	757	527	1607	6604	100.00
Frequency (%)	12.63	13.64	6.90	5.72	3.74	13.58	11.46	7.98	24.33	100.00	

A total of 112 repeat motifs were identified among the complete SSRs, of which there were 2 mononucleotides, 8 dinucleotides, 30 trinucleotides, 24 tetranucleotides, 12 pentanucleotides and 36 hexanucleotides, respectively (Table 3). Although SSR repeat types from mononucleotide to hexanucleotide all existed and they were also abundant, their occurrence frequency was quite different. The proportion of mononucleotide repeats dominated by A/T type was the highest (2951, 44.68%), and then dinucleotide repeats dominated by AG/CT and GA/TC (2236, 33.86%), trinucleotide repeats dominated by GAA/TTC and CCA/TGG (1275, 19.31%) and hexanucleotide repeats (68, 1.03%). Tetranucleotide and pentanucleotide repeat motifs exhibited relatively low frequencies, accounting for 0.76% and 0.36% of the total motif types, respectively. The statistical analysis of all SSR loci showed that the 5 repeat motif types with the highest occurrence frequency were in order as follows: A/T (2816, 42.64%), AG/CT (807, 12.22%), GA/TC (636, 9.63%), AC/GT (234, 3.54%) and CA/TG (215, 3.26%) (Table 3).

**Figure 6:**
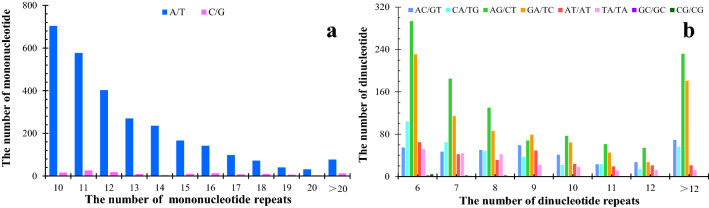
The types and numbers of mononucleotide and dinucleotide repeat motifs.

In *P. catalpifolia*, A/T was the most common mononucleotide repeat motif, accounting for 95.43% (2816) of all mononucleotide repeats, while C/G represented only 4.57% (135) (Fig. 6a). Of the dinucleotide repeats, AG/CT motif was the most frequent (807, 36.09%), followed by GA/TC (636, 28.44%), AC/GT (234, 10.47%) and CA/TG (215, 9.62%). The fewest dinucleotide motifs were GC/GC and CG/CG, each representing 1.79% (4) of the total dinucleotide repeats (Fig. 6b). There were 30 trinucleotide motifs present, of which GAA/TTC and CCA/TGG were the most frequent, accounting for 8.16% (104) and 8.00% (102) of the trinucleotide motifs, respectively, followed by AGA/TCT (82, 6.43%), CAG/CTG (81, 6.35%), AAG/CTT (78, 6.12%), CCG/CGG (78, 6.12%) and TCA/TGA (68, 5.33%). The fewest trinucleotide motifs were ACG/CGT (6, 0.47%), ACA/TGT (5, 0.39%) and GTA/TAC (2, 0.16%) (Table 3). Of the 24 tetranucleotide repeat motifs, CCCT/AGGG was the most frequent (8, 16%), followed by TGAA/TTCA (4, 8%) and GATT/AATC (4, 8%). The number of TTTG/CAAA, AAAT/ATTT, GAAA/TTTC, CTTT/AAAG and GGAG/CTCC all had 3 and accounted for 6%, 3 tetranucleotide motifs all with the number of 2 and another 13 repeat motif all with the number of 1. Within the 12 pentanucleotide repeat motifs, CCACC/GGTGG was the most frequent (9, 37.50%), followed by TCCTC/GAGGA (4, 16.67%) and CTTTT/AAAAG (2, 8.33%); the number of remaining 9 repeat motifs all were 1. Of the 36 hexanucleotide repeat motifs, AAGAGA/TCTCTT was the most frequent (8, 11.76%), followed by TTTCTT/AAGAAA (6, 8.82%). The number of 6 repeat motif types were all 3, 8 each were 2 and the remaining 20 each were 1 (Table 3).

## Discussion

The lack of reference genome has impeded basic genetic research in *P. catalpifolia* and its related species. However, SMRT sequencing technology can generate full-length transcript sequences without a reference genome^35–37^ and has been widely used to predict and validate gene models related to some unique traits in species^38^. In this study, we used the SMRT technique to perform full-length transcriptome sequencing in *P. catalpifolia* using PacBio RS II platform. In total, 28.83 Gb sequencing data were obtained including 349,745 full-length non-chimeric sequence reads, which was similar to the number of FLNC reads in *Rhododendron lapponicum*^39^. After subjecting the reads to clustering, error correction and redundant sequence removal, a total of 25,969 HQ transcripts were finally obtained. Very-long-read sequences were generated using the SMRT sequencing technology, and one read is considered a full-length transcript under normal circumstances^40^. The HQ transcripts generated using SMRT sequencing were longer in length than those generated using an Illumina system. In this study, the average length of the HQ transcripts in *P. catalpifolia* was 1624 bp, while the mean unigene length was 945 bp in tung tree^41^, 683 bp in *Pueraria lobata*^42^ and 690 bp in *Eucommia ulmoides*^43^, each of which were sequenced using an Illumina system. In addition, we found that HQ transcripts > 1000 bp in length accounted for 84.04% of all HQ transcripts in our research, which was much higher than that in *P. australis* (40.09%)^44^ and *P. tomentosa* (42.16%)^45^ using Illumina sequencing technique. Our results demonstrated that SMRT sequencing is a reliable and efficient method to obtain full-length transcript sequences in species without an annotated reference genome.

We annotated 25,602 HQ *P. catalpifolia* transcripts using five public databases. The annotated HQ transcripts accounted for 98.59% of all HQ transcripts, a similar rate to those of transcriptomics studies in *R. lapponicum*^39^ and *Medicago sativa*^46^. The 367 HQ transcripts with no predicted functions are likely to be species-specific or unknown genes in *P. catalpifolia*. GO classification of the HQ transcripts indicated that the majority were associated with the GO terms metabolic processes, binding, catalytic activity, cellular processes, cell and cell part. HQ transcript annotation using KOG indicated that a large number of transcripts were involved in posttranslational modifications, protein turnover, chaperones, translation, and ribosomal structure and biogenesis. A total of 13,829 HQ transcripts were assigned to specific KEGG pathways, such as carbohydrate metabolism, energy metabolism, translation, folding, sorting and degradation. We also found that many HQ transcripts exhibited multiple molecular functions and participated in diverse biological pathways. Our study provides a wealth of genetic information for molecular research into the growth and development of *P. catalpifolia* leaves, particularly in response to drought stress.

In recent years, SSR molecular markers have been widely used for genetic map construction, genetic diversity analyses and functional gene mining. However, the traditional methods of SSR primer development are time-consuming, complex and costly, thus hindering their development seriously. While the SSR primers developed on the basis of transcriptome sequencing data information are economical, efficient, and abundant, which has gradually become one of important methods. Furthermore, SSR molecular markers are rapidly being developed alongside recent advancements in transcriptome sequencing technology^47,48^. In our study, a total of 7367 SSR loci were detected from 25,969 HQ transcripts, including 763 complex SSRs and 6604 complete SSRs. The frequency of the SSRs was 28.37%, and the average distribution distance was 5.59 kb. Among the 6604 complete SSRs, the most abundant and frequent mononucleotide, dinucleotide and trinucleotide motifs were A/T, AG/CT and GAA/TTC, respectively; studies examining SSRs in *Hevea brasiliensis*^49^, Chinese cabbage^50^ and *R. lapponicum*^39^ produced similar results. A/T was the most abundant mononucleotide motif (2816, 95.43%), which was consistent with a study performed by Lagercrantz et al^51^. AG/CT (807, 36.09%) and GA/TC (636, 28.44%) were the most abundant dinucleotide motifs, and CT repeats usually existed in transcriptional regions that might take part in antisense transcription and have an effect on gene regulation^39,52^. There were differences in SSR abundance of different plant species in diverse researches, and repeat number of 6, 10, 5, 11, and 12 occupied 59.30% of the total complete SSR loci in our study. The SSR markers that we have developed in this work will facilitate mining for drought resistance genes, breeding drought resistant varieties, genetic diversity analyses and genetic map construction in *P. catalpifolia*. Of course, the SSRs found in this study were predicted theoretically and should be verified experimentally before further using.

## Materials and methods

### Plant materials and RNA extraction

*P. catalpifolia* seedlings were planted in separate pots at Mengzhou Forest Farm at the Paulownia Research and Development Center of State Administration of Forestry and Grasslands (Jiaozuo, Henan, China, 112° 42′ 58″ E, 34° 51′ 38″ N). The third and fourth fully expanded functional leaves from the top of the stem were collected at 0, 8 and 16 days after drought stress, respectively. The leaves were immediately frozen in liquid nitrogen and stored at − 80 °C until the experiment^23^. The *Paulownia catalpifolia* used in this study were identified by Paulownia Research and Development Center of State Administration of Forestry and Grassland, and the collection and use of *Paulownia catalpifolia* samples in our experiment comply with the guidelines of Paulownia Research and Development Center of State Administration of Forestry and Grassland. Total RNAs extraction were performed using the EZ-10 DNAaway RNA mini-prep kit (Sangon Biotech Co., Shanghai, China) following the manufacturer’s instructions. The total RNAs of three samples above were mixed equally according to the method of Diao^53^ to form the sample S for transcriptome sequencing. The degrees of RNA degradation and contamination were evaluated using 1% agarose gels^39^. The RNA purity and concentration were checked using the NanoPhotometer spectrophotometer (Implen, CA, USA) and Qubit RNA Assay Kit (Life Technologies, CA, USA), respectively^22^. RNA integrity was analyzed using an Agilent Bioanalyzer 2100 system (Agilent Technologies, CA, USA)^22^. The resulting high-quality RNA was used for full-length transcriptome sequencing.

### cDNA library construction and SMRT sequencing of the full-length transcriptome

Full-length cDNA was synthesised from 1.0 μg purified mRNA using the SMARTer PCR cDNA Synthesis Kit (Clontech, USA) according to the manufacturer’s protocol, its size were selected using the BluePippin Size-Selection System (Sage Science, USA) and then PCR amplified again. The cDNA library was constructed after repairing the ends, connecting dumbbell-shaped SMRT adapters, performing exonuclease digestions and conducting a secondary screening using BluePippin. After the cDNA library had passed quality control using the Qubit 2.0 and Agilent 2100, full-length transcriptome sequencing of *P. catalpifolia* was performed using the PacBio RS II platform, based on the target data volume^23^.

### Quality control and functional annotation of the full-length transcriptome

The raw SMRT data were pre-processed using the SMRT Pipe analysis workflow within the PacBio SMRT Analysis software suite. Examination of the polyadenosine signal and 5′ and 3′ adaptors, as well as error correction, were performed following the methods similar to the one described^54^. Full-length SMRT transcripts were identified, and non-redundant HQ transcripts were acquired using CD-HIT-EST software^55^. Clustering and removal of redundant sequences were performed using the PacBio SMRT LINK Cluster tool, and all HQ transcripts were aligned to nucleotide and protein databases using BLASTX^54^. The databases used in this study were NCBI non-redundant, gene ontology (GO), eukaryotic orthologous groups (KOG), Kyoto encyclopedia of genes and genomes (KEGG) and Swiss-Prot.

### Identification of lncRNAs, coding sequences (CDSs) and AS variants

LncRNA candidates were identified using the following software: coding potential calculator 2 (CPC2), coding potential assessment tool (CPAT), predictor of long non-coding RNAs and messenger RNAs based on an improved *k*-mer scheme (PLEK), and the coding–non-coding index (CNCI), respectively. LncRNAs with > 200 nucleotides were selected. TransDecoder version 3.0.0 was used to identify candidate coding sequences (CDSs) in the full-length transcriptome of *P. catalpifolia*. All non-redundant HQ transcripts were aligned using a previously described method^56^. Candidate AS events were identified using the selection criteria described by Diao et al.^53^.

### Identification and characterization of SSRs

The microsatellite identification tool (MISA) was used to identify SSRs within the 25,969 HQ transcripts, and the characteristics of the repeated motif types were further analyzed statistically. In this study, the SSR locus were identified according to the criteria below: the repeat number of mononucleotide motifs was ≥ 10 and the repeat numbers of di-, tri-, tetra-, penta- and hexanucleotide motifs were ≥ 6, 5, 5, 5 and 5, respectively.

## Data Availability

The raw data from SMRT sequencing are accessible at NCBI under bioproject (PRJNA565572).
